# Public health systems strengthening in Africa: The role of South Africa Field Epidemiology and Laboratory Training Programme

**Published:** 2011-12-14

**Authors:** Lazarus Kuonza, Khin San Tint, Bernice Harris, Immaculate Nabukenya

**Affiliations:** 1 National Institute for Communicable Diseases, Private Bag X4, Sandringham 2131, South Africa; 2 African Field Epidemiology Network P.O. Box 12874, Kampala, Uganda

**Keywords:** Field Epidemiology and Laboratory Training Program, South Africa, public health, outbreak, surveillance, International Health Regulations

## Abstract

The South Africa Field Epidemiology and Laboratory Training Programme (SAFELTP) was created in 2006 after recognizing the need to build and sustain the country's human resource capacity in field (applied) epidemiology and public health practice. The programme was formed as a collaboration between the South Africa Department of Health (DoH), the National Institute for Communicable Diseases (NICD), the National Health Laboratory Services (NHLS), the US Centers for Disease Control and Prevention (CDC) and the University of Pretoria. The primary goal of the programme was to produce field-trained epidemiologists equipped with knowledge and practical skills to effectively and efficiently address the public health priorities of South Africa. SAFELTP is a 2-year full-time training, consisting of a combination of classroom-based instruction (30%) and mentored field work (70%). The training places emphasis on public health surveillance, investigation of disease epidemics, public health laboratory practice and communication of epidemiologic information, among other aspects of epidemiology research. At completion, residents are awarded a Master of Public Health (MPH) degree from the University of Pretoria. Since its inception in 2006, 48 residents have enrolled onto the programme and 30 (62%) of them have completed the training. Over the past 5 years, the residents have conducted more than 92 outbreak investigations, 47 surveillance evaluations, 19 planned studies, analyzed 37 large databases and presented more than 56 papers at local and international conferences. In recognition of the high-quality work, at least five SAFELTP residents have received awards at various international scientific conferences during the 5 years. In conclusion, the South Africa FELTP is now fully established and making valuable contributions to the country's public health system, albeit with innumerable challenges.

## Introduction

The South African Field Epidemiology and Laboratory Training Programme (SAFELTP) was created in 2006 as a collaboration between the South Africa Department of Health (DoH), the National Institute for Communicable Diseases (NICD), the National Health Laboratory Services (NHLS), the United States (US) Centers for Disease Control and Prevention (CDC) and the University of Pretoria. The programme was formed after recognizing the need to build and sustain the country's human capacity in applied epidemiology and the need to foster collaboration between laboratory practice and epidemiology. The program started operation in 2007 and since then there has been a yearly intake. The program envisions a public health system that promotes wellness in communities through evidence-based decision-making, in line with other FELTPs around the globe [1,2]. The program hopes to achieve this vision by strengthening the epidemiological capacity among health professionals at all levels of the health system in South Africa through mentored competency-based training. The programme also aims to bridge the gap between the health departments and the laboratory services in the control of communicable and non-communicable diseases.

### Description of the program

The South Africa FELTP is physically located in Johannesburg within the National Institute of Communicable Diseases (NICD) of the National Health Laboratory Services (NHLS), and the program works closely with the national and provincial Departments of Health (DoH) and other partners. SAFELTP is an applied epidemiology and laboratory training programme that provides multi-disciplinary field-based and problem-oriented instruction. SAFELTP is a 2-year full-time training and service program, modeled after CDC′s renowned Epidemic Intelligence Service (EIS) [3,4]. Residents spend 30% of the time attending classroom-based courses and 70% performing mentored field duties. The training places emphasis on the application of epidemiological principles in public health surveillance, outbreak investigation, program evaluation, health data management and the role of the laboratory systems in epidemiology, among other public health areas. Graduates from the programme are equipped with hands-on investigative and analytic skills to identify public health problems, plan and execute field-based scientific research, design and recommend effective solutions to the problems, and communicate the solutions to the relevant professionals. At completion, residents receive a Master of Public Health (MPH) from the University of Pretoria.

Field placement sites consist of Provincial DoHs and different programmatic areas within National DoH, NICD, or NHLS and pharmacovigilance unit of the Medical University of Southern Africa (MEDUNSA). In order to accommodate the needs of the fellows and the respective nominating institutions, fellows are placed with their representative institution whenever possible. During the field training, residents acquire practical skills in applied epidemiology while at the same time providing epidemiological services to the field placement site (DoH, NHLS, etc). The SAFELTP secretariat maintains regular monitoring and appraisal of the field sites to ensure that individual needs of the trainee and the broader objectives of the field placement are met.

One of the aims of SAFELTP is to increase collaboration and strengthen linkages between epidemiologists and the public health laboratorians. Residents who enroll on to the SAFELTP residency program choose one of two areas of concentration, or “tracks": epidemiology track and laboratory track. Both tracks are 2 years long and most modules and field activities are shared between the tracks. SAFELTP is headed by a Program Director who is supported, technically, by epidemiology and laboratory track coordinators who manage the academic elements of the respective tracks, a field coordinator who oversees the field component of the training, field epidemiologists who provide mentorship to residents and an administrative team that is responsible for the administrative aspects of the program. The overall running of the program is guided by an Advisory Committee made up of partners and experts from the epidemiology community in South Africa and beyond.

### Basic epidemiology short courses

In addition to the 2-year residency program, SAFELTP also offers certificate awarding on-job training courses typically consisting of 2 week-long didactic workshops separated by a 2 to 4 month field project to apply field epidemiology principles to a local public health need. The courses are targeted towards health professionals involved in communicable disease control, disease surveillance, outbreak investigations and data management at various levels of the health system, and can be tailored to meet the specific needs of the requesting institution. Relationships within the FELTP

The programme received seed funding from the US government through the President′s Emergency Plan for AIDS Relief (PEPFAR) funds. In country, the programme has received financial and in-kind support from various partners, including the Department of Health (DoH), the University of Pretoria, the Department of Agriculture (DoA) and the NHLS. The national and provincial DoH and DoA send their officials who are responsible for surveillance, monitoring and evaluation of priority programs into the SAFELTP with 2 years study leave and after completion of the training retain the trained officials within the system. The departments agree to be the field placement sites, to provide co-supervision of the residents during field activities and projects, to facilitate the participation of residents in outbreak investigation and access to health datasets that belong to the departments. The School of Health Systems and Public Health of the University of Pretoria provides academic support and conducts some of the didactic teachings on public health related modules, biostatistics and demography. The director of the programme is permanently employed by the NICD. During the first three intakes the NHLS sponsored four residents onto the 2-year residency programme per intake. Participation in epidemiology networks

SAFELTP is affiliated to international networks of related training programs around the world, notably the regional African Field Epidemiology Network (AFENET) and the global Training Programs in Public Health Interventions Network (TEPHINET). The networks provide trainees with opportunities to share experiences and expertise with trainees from other parts of the world, through conferences and other information sharing platforms. The networks also provide resources for various FELTP core-activities.

South Africa FELTP was the second programme in Africa to include the laboratory component. Consequently the programme is proud to have provided technical assistance to other African FELTPs that subsequently established laboratory tracks namely, programmes in Nigeria, Rwanda and Mozambique.

In December 2010 South Africa FELTP hosted the 6th Global TEPHINET Scientific Conference in Cape Town. The conference was attended by more than 500 delegates from 48 countries, and 249 abstracts (99 oral presentations and 150 posters) were presented. SAFELTP staff and residents played an integral role in the preparation and hosting of the conference.

## Some achievements by the Program

As of 2011 the program has enrolled five cohorts (a total of 48 residents). The first three cohorts have completed FELTP residency to date (totaling thirty fellows), and 19 have formally graduated from the University of Pretoria (Table 1).

**Table 1:** South Africa Field Epidemiology and Laboratory Training Programme enrolment and graduation rate, 2007-2011

Cohort	Number enrolled	Number graduated from university	Graduation rate
2007	10	5	50%
2008	9	6	67%
2009	11	8	73%
2010	9	Still in training	-
2011	9	Still in training	-

Of the 30 graduates that have completed FELTP training to date, 24 (80%) have been absorbed within the public health sector in South Africa, mostly within the Departments of Health (11) and the National Health Laboratory Services (12) (Table 2). The programme has also trained two fellows from West Africa (Togo and Burkina Faso). These have returned to their home countries and have also been re-absorbed within the respective public health systems.

**Table 2:** Employment positions of graduates of the South Africa Field Epidemiology and Laboratory Training Programme

Institution category	No of graduates	
	(n=30)	%
Provincial Departments of Health	8	27%
National Institute for Communicable Diseases (NICD)*	4	13%
National Health Laboratory Services (NHLS)*	3	10%
South Africa FELTP	3	10%
National Department of Health	2	7%
Private Research Organizations	2	7%
National Institute for Occupational Health (NIOH)*	2	7%
Ministry of Health (West Africa)	2	7%
Local Authority health department	1	3%
Further studies	1	3%
Department of Agriculture	1	3%
Private business	1	3%

* NICD and NIOH are units within the NHLS

FELTP residents and graduates have made valuable contributions in strengthening the response of the various units to disease outbreaks and have also impacted positively on functioning of the surveillance systems and public health programs in their places of work. Among the graduates that are working within the DoH and the DoA, six have been elevated to senior management levels and are making significant contributions to policy decisions within their departments.

Within the NHLS structures FELTP graduates have been absorbed into the critical epidemiologist positions within the different units and have made significant contributions to the epidemiological analysis of laboratory data and have also been involved in key field investigations throughout the country. Thus these FELTP graduates have made significant in-roads in strengthening the public health laboratory function of the National Health Laboratory Services (NHLS), one of the goals at the inauguration of the FELTP.

## Major public health contributions

### Role during the 2010 FIFA world Cup

In June 2010 during the Fédération Internationale de Football Association (FIFA) World Cup competitions held in South Africa, FELTP staff, residents and graduates played a key role in supporting the national and provincial Departments of Health, NHLS and NICD, in establishing and running a national surveillance system for epidemic prone health conditions and responding to disease outbreaks related to the monumental soccer event. FELTP residents coordinated the collection, collation and epidemiological analysis of surveillance data from centres across the country and took park in the investigation and response to disease outbreaks detected through the surveillance activities. Prior to the World Cup event, the program conducted two short course trainings for provincial health officials, focusing on strengthening surveillance and outbreak response during the event.

### Major public health contributions: Role in implementation of the International Health Regulations (Table 3)

According to the International Health Regulations 2005 (IHR 2005), countries should strengthen their core capacity for disease surveillance and public health response systems in order to effectively prevent and control the international spread of diseases [5]. Strengthening disease surveillance and public health response are the primary goals of FELTPs, hence the IHR 2005 underscores a need to build and sustain FELTPs, if countries are to make better contributions to global health capacity [6]. In South Africa the FELTP has played a huge role in building the human resource capacity to enhance the implementation of the IHR 2005. In addition to the core curriculum, FELTP staff has assisted the Communicable Diseases Control Directorate of the National DoH in reporting the assessment of national core capacities of the implementation of the International Health Regulations (IHR 2005). On behalf of the University of Pretoria the programme staff have also taken part in trainings on the implementation of the IHR 2005, which are coordinated by the World Health Organization.

**Table 3:** Summary of key achievements of the South Africa Field Epidemiology and Laboratory Training Programme for the last five years

Activity	Frequency
Outbreaks investigations and response	92
Surveillance systems evaluated	47
Research studies done	19
Large data bases analyzed	37
Scientific presentations at conferences	56
Publications by the trainees	26
Short courses conducted	11

## Growing demand for the program

The demand for enrolment onto the program has significantly grown over the years. Within South Africa the number of people applying for enrolment onto the program has grown from 15 applications in 2007 to more than 40 in 2010 and 2011. Over the past 2 years, the program has also received applications from health professionals in neighboring countries (Botswana, Swaziland and Malawi) and from as far as Liberia in western Africa.

The growth and expansion in the FELTP has also been seen through the growing number of field placement sites for FELTP residents. The programme has established placement sites with DoHs in all the nine provinces, a number of directorates within the national DoH, with local authorities in some urban cities, and within units in the NICD and with some semi-private research organizations. The program has also received a growing number of requests from different organizations to have FELTP residents placed with them. This evidently indicates a rising appreciation of the role of FELTPs within the health system in South Africa.

The programme has also observed an increase in requests for the basic epidemiology short courses. Some provinces are now paying the total costs of the trainings, with the programme staff providing the training. This shows a greater appreciation of the role of the short courses within the health system whereas the FELTP would previously pay for all the costs in running the courses.

## Challenges in implementing the South Africa FELTP

Strong linkages with universities, health departments and international agencies are critical in building a sustainable FELTP [2,7]. In South Africa the FELTP has received inadequate institutional support from key authorities within the National DoH. This has compromised the sustainability of the programme in a number of ways. Firstly, it has been a challenge for the FELTP to enroll key professionals from the DoHs. The current human resource development policies do not support a 2 year paid study leave for professionals working within the department. This has forced some professionals to resign from their posts to come onto the program, and has compelled some good potential fellows to turn down the offer for training. Such a situation then works against the whole goal of a FELTP, which is to strengthen human resource capacity within the public health system.

Secondly, the FELTP is geographically placed within the NICD, which is considered by most institutions to be a laboratory establishment. This set-up, coupled with weak linkages with the DoH, has often made it difficult for the FELTP to attract key health professionals, particularly physicians, who are not within the laboratory system. For any FELTP to be sustainable academically and financially, the training programme should be incorporated within larger health department structures for human resource planning and development as well as structures for financial planning and budgeting [2,7].

Thirdly, there are no clear career pathways for FELTP graduates within the DoH structures, and the roles that FELTP graduates can play within the health system are not clearly defined. This often results in frustrations among graduates who feel that they are not granted opportunities to apply the skills acquired during FELTP residency. A few FELTP graduates have left the public health system to join private research organizations where they feel their skills will be more useful.

Having dedicated and competent staff at the FELTP secretariat is critical in building a sustainable high-quality FELTP programme. In South Africa the majority of the posts at the FELTP secretariat offer renewable yearly contracts, and the FELTP has experienced challenges in attracting experienced personnel (public health laboratory specialists and epidemiologists) to fill some of the posts. Most similar institutions offer favorably longer contracts or permanent posts. This compromises the mentoring of residents during field placement; the available staff become too stretched to provide the robust mentorship that FELTP trainees require. This is worsened by the limited technical expertise among field supervisors in some field placement sites.

The FELTP continues to make efforts to engage the various levels of the DoH to rectify these challenges. The programme is in the process of lobbying the DoH so that the FETP can be included in the department's human resources development strategy.

## Conclusions

In conclusion, the South Africa FELTP is now fully operational and is making valuable contributions to the country's public health system, albeit with innumerable challenges. The programme is unique in that its secretariat is physically and functionally detached from the structures of the health department. As a result, there has been limited engagement of key stakeholders within the health department, and inevitably this has compromised the growth of the programme. The main lesson that can be learnt from the South Africa situation is that establishing strong linkages with the ministry of health is crucial in building a sustainable high-quality FELTP.

In moving forward, the South Africa FELTP should come up with a broad advocacy strategy, primarily targeting key decision makers within the national DoH, to increase the visibility of the FELTP. The goal should be to advocate for FELTP to be incorporated into the health department's strategic plan for workforce development. As a precursor to the advocacy strategy, the FELTP should consider conducting a comprehensive sustainability analysis in order to streamline the future operations of the programme. This is especially important taking into consideration the constantly decreasing funding for the FELTP activities. The analysis will also look at strategies to reduce programme related costs, while maintaining the quality of the training.

As part of the advocacy, the programme should put together an all-inclusive marketing tool describing the current and future activities of the programme and outlining how current and potential partners can be incorporated. The FELTP should also strengthen the marketing of the programme in order to attract high-quality residents. Targeted marketing of certain professional groups (e.g., physicians) should be employed. Initially funds will be required to attract the physicians onto the training (in the form of stipends), to match the registrar packages available at various local universities for clinical specialties. When the department of health becomes more engaged, the programme can then lobby for similar registrar posts to be created for physicians who come onto the FELTP.

To support the advocacy efforts, the FELTP should put more effort in showcasing the work done by residents during training, in the form of publications in peer-reviewed journals, presentations at national forums, etc. This will enhance the visibility of the programme. Lastly, the programme should come up with an objective monitoring and evaluation plan which will ensure continuous quality improvement.
